# Plet1 is an epigenetically regulated cell surface protein that provides essential cues to direct trophoblast stem cell differentiation

**DOI:** 10.1038/srep25112

**Published:** 2016-04-28

**Authors:** Alexander Murray, Arnold R. Sienerth, Myriam Hemberger

**Affiliations:** 1 Epigenetics Programme, The Babraham Institute, Babraham Research Campus, Cambridge CB22 3AT, UK; 2 Centre for Trophoblast Research, University of Cambridge, Downing Street, Cambridge CB2 3EG, UK

## Abstract

Gene loci that are hypermethylated and repressed in embryonic (ESCs) but hypomethylated and expressed in trophoblast (TSCs) stem cells are very rare and may have particularly important roles in early developmental cell fate decisions, as previously shown for *Elf5*. Here, we assessed another member of this small group of genes, *Placenta Expressed Transcript 1* (*Plet1*), for its function in establishing trophoblast lineage identity and modulating trophoblast differentiation. We find that *Plet1* is tightly repressed by DNA methylation in ESCs but expressed on the cell surface of TSCs and trophoblast giant cells. In hypomethylated ESCs that are prone to acquire some trophoblast characteristics, Plet1 is required to confer a trophoblast-specific gene expression pattern, including up-regulation of *Elf5*. *Plet1* displays an unusual biphasic expression profile during TSC differentiation and thus may be pivotal in balancing trophoblast self-renewal and differentiation. Furthermore, overexpression and CRISPR/Cas9-mediated knockout in TSCs showed that high Plet1 levels favour differentiation towards the trophoblast giant cell lineage, whereas lack of Plet1 preferentially induces syncytiotrophoblast formation. Thus, the endogenous dynamics of *Plet1* expression establish important patterning cues within the trophoblast compartment by promoting differentiation towards the syncytiotrophoblast or giant cell pathway in Plet1-low and Plet1-high cells, respectively.

Cells of the placental trophoblast lineage are the first to differentiate after fertilisation when they are irrevocably set aside from all other cells that will form the embryo proper as well as other extraembryonic structures. This first cell fate decision event is directed by a handful of critical transcription factors that are induced in individual blastomeres dependent on their position, extent of polarisation and number of cell-cell contacts^1^,^2^,^3^,^4^. While the epigenome must establish a permissive environment for these initial lineage decisions to occur, the main role of DNA methylation is to reinforce the commitment of cells to their respective fate after the lineages have been established by the blastocyst stage, thereby firmly ‘locking in’ lineage fate^5^,^6^.

Factors that contribute to confer stable cell lineage commitment can be particularly well studied in stem cells derived from the mouse blastocyst-stage embryo, notably embryonic stem cells (ESCs) derived from the inner cell mass and epiblast, and trophoblast stem cells (TSCs) derived from the trophectoderm (TE) and post-implantation extraembryonic and chorionic ectoderm. ESCs that are globally hypomethylated due to genetic deficiency of *Dnmt1*, *Dnmt3a*/*3b*, or *Uhrf1* have the ability to “trans-differentiate” into the trophoblast lineage from which they are normally excluded^5^,^7^,^8^. Since this scenario implies that loss of methylation at specific loci enables a widening of developmental potential, our focus has been in particular on genes that are hypomethylated and expressed in TSCs, but hypermethylated and repressed in ESCs. Overall, this specific pattern of differential methylation is very rare, perhaps suggesting that the affected genes are particularly important for early cell fate commitment. Indeed, in earlier studies this approach had identified the transcription factor Elf5 that we found is most stringently regulated at the epigenetic level, reinforcing trophoblast fate and TSC potential in the trophoblast lineage, but abrogating this pathway in ESCs through tight repression by DNA methylation^5^. Refinement of the resolution of the DNA methylation landscape through recent advances in sequencing technology has expanded this group of so-called lineage “gatekeepers” to 10 genes that are differentially methylated and expressed in a pattern like *Elf5*, i.e. hypomethylated and expressed in TSCs but hypermethylated and repressed in ESCs, in a consistent and robust manner^9^.

Among these additional putative “gatekeeper” genes was the *Placenta Expressed Transcript 1* (*Plet1*) locus that caught our attention for a potential role in cell lineage specification and defining stem cell potency because of its known expression pattern in the presumptive TSC niche *in vivo* and its rapid up-regulation in *Dnmt1*^−/−^ ESCs upon induction of trans-differentiation^9^,^10^. Plet1 encodes a post-translationally modified protein, possessing a glycosylphosphatidylinositol (GPI) anchor, as well as N-linked carbohydrates^11^, indicative of membrane localisation. The gene was first identified through the analysis of EST data as a factor strongly expressed in the placenta^12^. *In situ* hybridisation on E5.5-E8.0 conceptuses demonstrated a highly restricted expression pattern of *Plet1* in the distal-most region of the extraembryonic ectoderm (ExE) directly overlying the epiblast and later in the chorionic ectoderm, i.e. structures known to harbour TSC progenitor cells^13^,^14^. While ExE cells further away from the epiblast do not express Plet1, expression is again observed in ectoplacental cone (EPC) cells, and also from E7.5 onwards within the embryo itself in the node^10^,^15^.

Apart from its expression during embryogenesis, Plet1 has been reported to mark distinct populations of progenitor cells in the thymic epithelium, in hair follicles, in mammary gland and prostate epithelia, the salivary gland and in the major duct epithelium of the pancreas, overall pointing to an important role for Plet1 in epithelial stem and/or progenitor cell types^11^,^16^,^17^,^18^,^19^,^20^,^21^.

The compelling expression pattern in extraembryonic tissues of early conceptuses combined with our identification of *Plet1* as a gene under tight epigenetic control, akin to the transcription factor Elf5 that had previously been found to play an instrumental role in cell fate commitment and establishment of the TSC niche^5^,^22^, prompted us to investigate the function of Plet1 in the TSC compartment and in cell lineage maintenance in more detail. We find that although Plet1 alone is not sufficient to induce a cell fate switch between ESCs and TSCs, it is essential for the activation of key components of the trophoblast lineage, including *Elf5*. Within the trophoblast compartment Plet1 levels are associated with defining TSC fate and correctly allocating cells to the appropriate trophoblast sub-lineage; thus, differentiation of Plet1-negative trophoblast cells is skewed towards syncytiotrophoblast (SynT) whereas high Plet1 levels promote differentiation towards the trophoblast giant cell (TGC) pathway.

## Results

### Plet1 is differentially methylated and expressed between ESCs and TSCs

**Figure 1:**
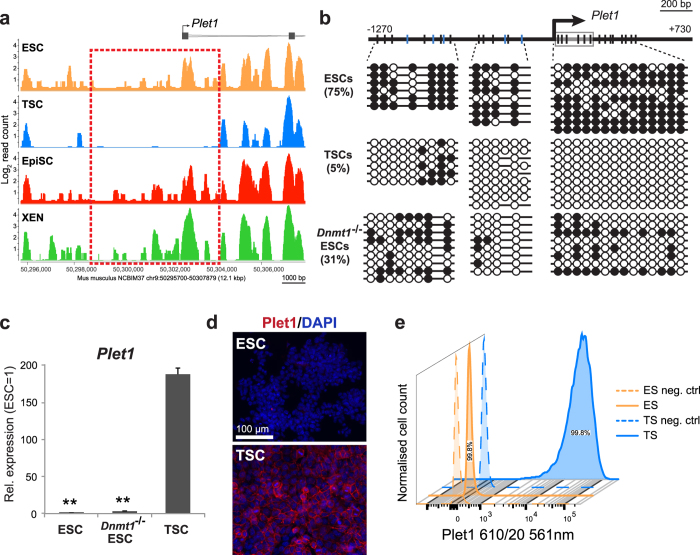
*Plet1* is differentially methylated and expressed between ESCs and TSCs. (**a**) DNA methylation-sequencing^30^ screen identifies differential methylation at the *Plet1* promoter which is hypermethylated in ESCs as well as in epiblast-derived stem cells (EpiSCs) and in extraembryonic endoderm stem (XEN) cells, but hypomethylated in TSCs. Each data track represents the mean of two independent cell lines. The differentially methylated region is indicated by the red dashed box. **(b)** Bisulphite sequencing analysis of the *Plet1* promoter and first exon (box) and intron regions displays extensive methylation of CpG dinucleotides (filled circles) in ESCs compared with widespread hypomethylation (open circles) in TSCs, and intermediate DNA methylation levels in *Dnmt1*^−/−^ ESCs. Note that the 4th, 6th, 8th, 13th, and 15th CpG sites (indicated in blue) are polymorphic. **(c)** RT-qPCR analysis reveals significantly higher levels of *Plet1* expression in TSCs compared with ESCs. *Plet1* primers used were common to all isoforms. Data are mean of three replicates normalised to *Sdha*, *Pgk1* and *Tbp* and are displayed ± S.E.M. (**P < 0.005 against TSCs). **(d)** Immunofluorescence staining (without permeabilisation) of ESCs and TSCs showing high levels of Plet1 (red) only in TSCs. Nuclei were counter-stained with DAPI. **(e)** Flow cytometry analysis confirms differential expression levels of Plet1 protein between ESCs and TSCs. ESCs display a similar signal intensity to negative (no antibody) controls. Peak height is normalised and a bisector gate at 10^3^ splits the histogram into two parts with the percentages of cells in the corresponding peak region indicated. For each sample, a total of 20,000 cells were analysed.

DNA methylation profiling (meDIP-seq) approaches of stem cells of the early embryo had indicated that the promoter of the orphan protein Plet1 is hypermethylated in ESCs, epiblast stem cells (EpiSCs) and extraembryonic endoderm stem (XEN) cells, but hypomethylated in TSCs (Fig. 1a). To validate the meDIP-seq data, we performed bisulphite sequencing on three consecutive regions spanning the *Plet1* promoter and first exon and intron, which confirmed differential methylation between ESCs and TSCs with average CpG methylation values of 75% and 5%, respectively (Fig. 1b). ESCs deficient for the maintenance DNA methyltransferase Dnmt1 exhibited intermediate methylation levels at 31% across the *Plet1* locus (Fig. 1b), a situation that is very much akin to that observed at another key differentially methylated gene, *Elf5* (ref. 5).

The methylation differences of *Plet1* between ESCs and TSCs were inversely correlated with expression, as shown by semi-quantitative RT-PCR (RT-qPCR) analysis that revealed extremely low or virtually absent *Plet1* transcript levels in wild-type ESCs and comparatively high expression in TSCs (Fig. 1c). As with *Elf5*, the reduced levels of DNA methylation at the *Plet1* locus in *Dnmt1*-deficient ESCs *per se* did not lead to a significant up-regulation of *Plet1* expression when these cells are grown in ESC conditions (Fig. 1c; Supplementary Fig. S1a). Reflecting the differential abundance of transcript levels, immunofluorescence staining demonstrated a strong signal in TSCs but absence of Plet1 protein in ESCs (Fig. 1d). Furthermore, the differential expression of Plet1 was also shown by flow cytometry, which indicated localisation of at least some amount of Plet1 protein on the cell surface of TSCs (Fig. 1e; Supplementary Fig. S1b).

### Plet1 is necessary to induce trophoblast-like characteristics

**Figure 2:**
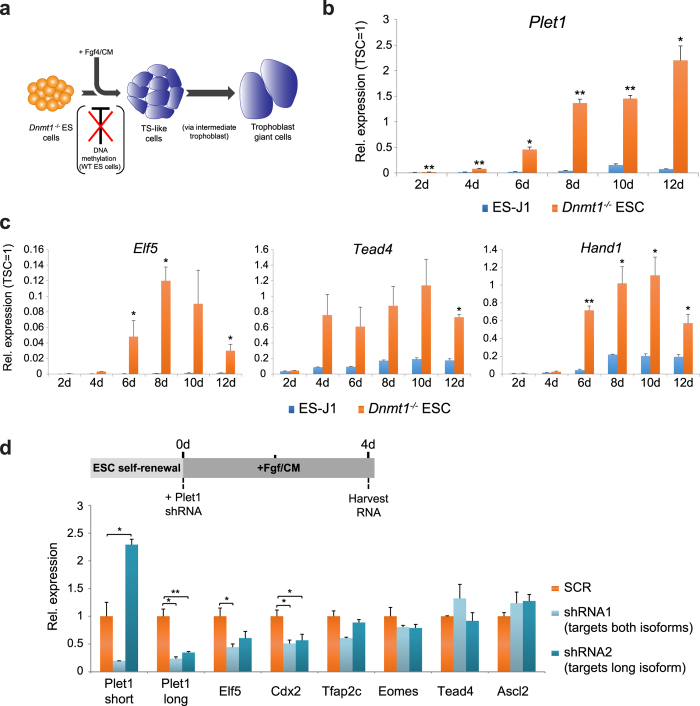
*Plet1* is up-regulated in methylation-deficient ESCs and required for the activation of key trophoblast genes. (**a**) Schematic displaying the trans-differentiation model of *Dnmt1*^−/−^ ESCs towards a trophoblast-like phenotype. (**b**) *Plet1* and (**c**) trophoblast marker gene expression was activated in *Dnmt1*^−/−^ ESCs during culture in TSC medium (+Fgf/CM) for 2–12 days. Data are displayed against expression levels in TSCs which are set to 1 after normalisation against housekeeping genes *Sdha* and *Pgk1*, and are the mean of three biological replicates ±S.E.M. (*P < 0.05, **P < 0.005). Please note, samples used are identical to those described in ref. 5. (**d**) Upper: schematic displaying experimental protocol: at 0d *Dnmt1*^−/−^ ESCs were transfected with shRNA constructs targeting *Plet1* isoforms (shRNA1 targets both isoforms; shRNA2 targets only the long GPI-anchored isoform) and transferred into TSC medium. Transfected (GFP positive) cells were isolated by flow cytometry after four days of trans-differentiation. Lower: RT-qPCR analysis reveals that knockdown of *Plet1* prevents up-regulation of trophoblast markers, notably *Elf5* and *Cdx2*, as well as Hand1 at later stages of differentiation (Supplementary Fig. S2b). Level of expression was normalised to housekeeping genes *Sdha* and *Pgk1*. Data are mean of three replicates and displayed as ±S.E.M. (*P < 0.05, **P < 0.005).

To assess a potential stem cell lineage-reinforcing role of Plet1 in the transition between ESCs to TS-like cells we employed the model of *Dnmt1*-deficient ESCs, as we have done before^5^,^9^. Due to their hypomethylated status that enables activation of important lineage gatekeeper genes like *Elf5*, *Dnmt1*^−/−^ ESCs acquire some trophoblast-like characteristics when cultured under TSC conditions^5^,^8^, which encompasses the presence of fibroblast growth factor 4 (Fgf4) and embryonic feeder cell conditioned medium (CM) (Fig. 2a). Indeed, similar to other trophoblast genes, *Plet1* expression was strongly up-regulated in a trans-differentiation time course of *Dnmt1*^−/−^ ESCs (Fig. 2b,c). This transcriptional up-regulation correlated with an increase in the proportion of Plet1-positive cells detected by flow cytometry even at early stages of the trans-differentiation process (Supplementary Fig. S1b). Despite the co-regulation with other trophoblast genes, however, we had previously shown that Plet1 alone is not sufficient to induce a TS-like fate from wild-type ESCs^9^. To investigate Plet1’s role in this cell fate transition in more detail, we here asked whether Plet1 is required for the induction of trophoblast characteristics from ESCs. For this purpose we performed transient (Fig. 2d) as well as stable transfection experiments (Supplementary Fig. S2) with two different shRNA constructs in *Dnmt1*^−/−^ ESCs targeting the main *Plet1* transcript isoforms. When cultured in ESC conditions, these shRNAs remained inconsequential as *Plet1* is not expressed. Upon shift to TSC conditions, however, the shRNAs suppressed the normal up-regulation of *Plet1* (Fig. 2d; Supplementary Fig. S2b). Importantly, this lack of *Plet1* expression abrogated the induction of other trophoblast genes normally up-regulated in *Dnmt1*^−/−^ ESCs, notably *Elf5* and *Cdx2*, as well as *Hand1* at later stages of differentiation (Fig. 2d, Supplementary Fig. S2b). Thus, although Plet1 is not sufficient to induce an ESC-to-TS-like cell fate transition, it is necessary for the activation of key components determining trophoblast cell fate.

### Plet1 expression dynamics in TSCs

**Figure 3:**
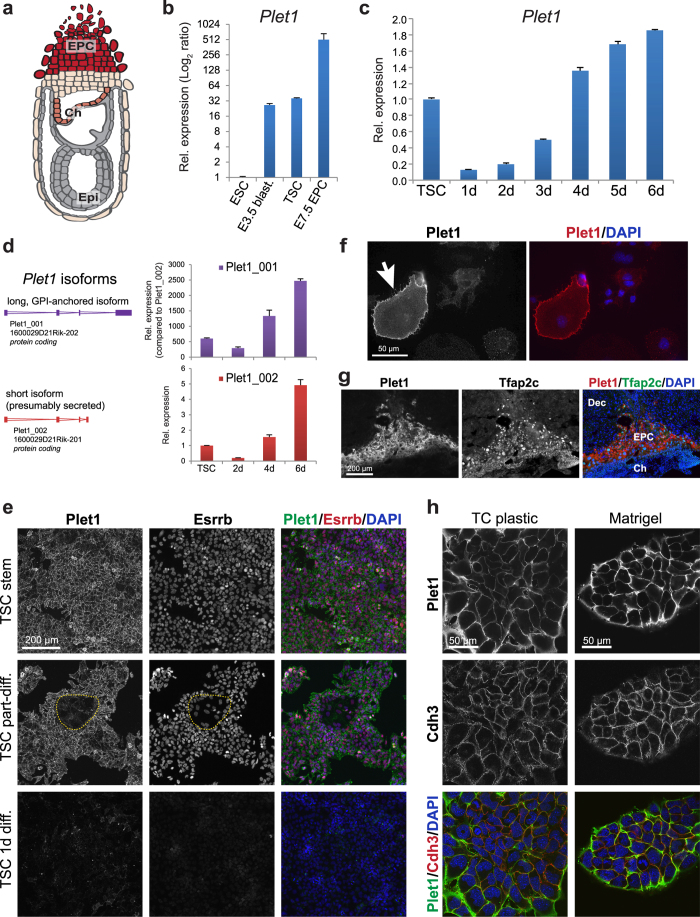
Dynamic regulation of *Plet1* expression with trophoblast differentiation. (**a**) Schematic diagram of post-implantation conceptus at E7.5, with grey and beige representing embryonic and trophoblast derived structures, respectively. The *in vivo Plet1* expression pattern in trophoblast^10^,^15^ is displayed by red shading, with intermediate expression levels in the trophoblast-derived chorion (Ch), low or absent expression in the intervening diploid trophoblast cells, and highest levels in the ectoplacental cone (EPC). (**b**) RT-qPCR analysis of *Plet1* in ESCs, E3.5 blastocyst, TSCs, and EPC. Expression was normalised to housekeeping genes *Sdha* and *Gapdh*; data are mean of three replicates (EPC, two biological replicates) ±S.E.M. **(c)** Relative expression levels of *Plet1* expression during a 6-day time course of TSC differentiation induced by withdrawal of Fgf/CM. The overall dynamics of *Plet1* expression levels are similar to the *in vivo* profile. Data represent the mean of six biological replicates ±S.E.M. (**d**) RT-qPCR analysis of the two annotated *Plet1* isoforms 001 (1846 bp) and 002 (623 bp). The overall expression pattern during TSC differentiation is similar for both isoforms, but the longer GPI-anchored isoform (001; purple) is expressed at a much greater level than the shorter isoform (002; red). Data are displayed relative to *Plet1-002* in TSCs and are mean of three biological replicates ±S.E.M.; housekeeping genes used were *Sdha* and *Hprt1*. (**e**) Immunofluorescence staining of TSCs in the stem cell state and upon early stages of differentiation (diff.) for Plet1 and the stem cell marker Esrrb. Plet1 staining intensity is abruptly down-regulated with the onset of differentiation (middle panel: Esrrb-low, partially differentiated TSCs highlighted by the dotted line; bottom panel: TSCs after 1 day of Fgf/CM withdrawal). Images in each channel are taken at identical exposure settings. (**f**) Immunofluorescence staining of differentiated TSCs for Plet1; the large, strongly Plet1-positive cell (arrow) is a trophoblast giant cell. (**g**) Immunofluorescence staining of an E8.5 mouse implantation site for Plet1, showing strong staining in the trophoblast giant cell layer. (**h**) Confocal images of double-immunofluorescence stainings for Plet1 and placental cadherin Cdh3 of TSCs grown on tissue culture plastic or in Matrigel reveal the close membrane association of Plet1.

In the early post-implantation conceptus *Plet1* is first expressed in the distal-most ExE in close proximity to the epiblast and slightly later in the chorion, both tissue layers where trophoblast cells with stem cell potential reside. However, strong *Plet1* expression is also detected in the EPC, some distance away from the ExE/chorion where more differentiated trophoblast cell types are located, while the intervening trophoblast cells are *Plet1*-negative (Fig. 3a)^10^. Indeed, we observed ~14-fold higher *Plet1* expression in E7.5 EPC compared to TSCs (Fig. 3b). We also assessed E3.5 blastocysts and detected expression levels similar to those observed in TSCs. Since *Plet1* is methylated and repressed in ESCs, it is tempting to speculate that the blastocyst expression arises from the TE layer from which TSCs are derived. Given the biphasic *Plet1* expression profile *in vivo*, we examined the dynamics of *Plet1* regulation during a TSC differentiation time course *in vitro*. Similar to the *in vivo* pattern, *Plet1* mRNA and protein expression was down-regulated at early stages of differentiation after 1–2 days, followed by a pronounced increase during subsequent days of TSC differentiation (Fig. 3c-e; Supplementary Fig. S3). These kinetics were profoundly different from other trophoblast stem and differentiation markers such as *Cdx2*, *Eomes*, *Ascl2*, *Gcm1*, *Pl1* (*Prl3d1*) and *Plf* (*Prl2c2*) that served as indicators of the progression of TSC differentiation (Supplementary Fig. S3a). The biphasic expression pattern was observed for both predicted isoforms of the *Plet1* gene (Fig. 3d), albeit at markedly different overall levels: the longer variant Plet1_001 (previously known as 1600029D21Rik-202), predicted to be GPI anchored, was by far the predominantly expressed isoform. By contrast, the shorter isoform Plet1_002 (1600029D21Rik-201) that lacks the GPI anchor and is presumably soluble and secreted, was expressed at comparatively much lower levels (Fig. 3d).

**Figure 4:**
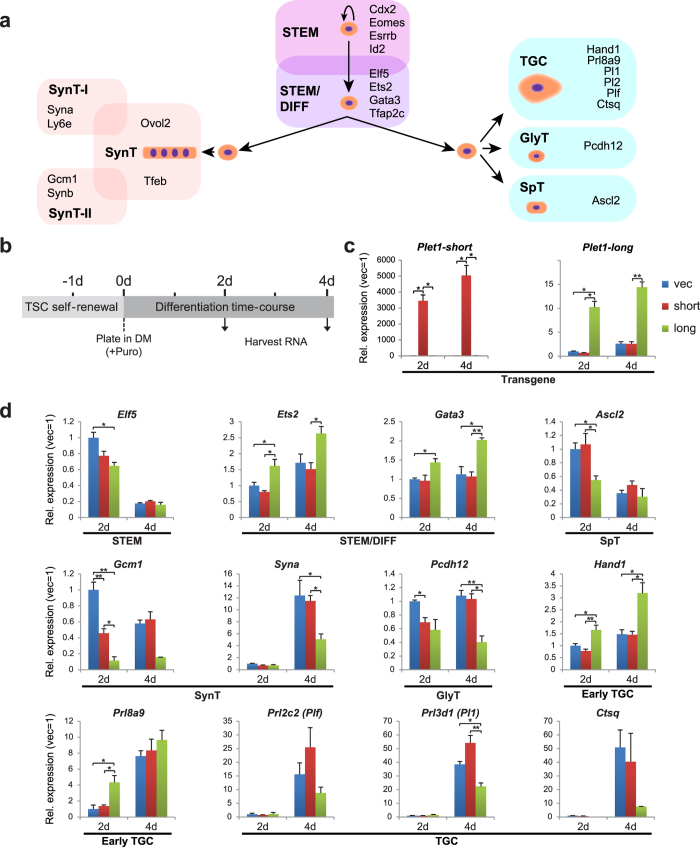
Overexpression of *Plet1* promotes TSC differentiation along the TGC trajectory. (**a**) Schematic diagram displaying the major trophoblast differentiation pathways and the marker genes indicative of the respective trophoblast cell types. TSC differentiation occurs along two major routes, either towards syncytiotrophoblast (SynT), or towards a spongiotrophoblast (SpT)-, glycogen cell (GlyT) and trophoblast giant cell (TGC) phenotype. We refer to this latter pathway generically as “TGC” trajectory due to the predominance of giant cell differentiation *in vitro*. (**b**) Schematic displaying experimental protocol: at −1d wild-type TSCs were transfected overnight with *Plet1* overexpression constructs (either short or longer GPI anchored isoform), and then (0d) plated in differentiation media (DM) with puromycin selection. Cells were harvested for RNA at 2 and 4d time-points. (**c**) Confirmation of successful *Plet1* isoform over-expression as analysed by RT-qPCR. (**d**) Effect of *Plet1* isoform over-expression on TSC and differentiated trophoblast markers. Data were normalised to *Sdha* and *Pgk1*, and are mean of three biological replicates ±S.E.M. (*P < 0.05, **P < 0.005).

This expression pattern was also confirmed by immunofluorescence staining; in TSCs cultured in stem cell conditions, frequent co-expression of Plet1 with TSC markers such as the transcription factor Esrrb^23^ was observed, while flattened clusters of slightly larger and Esrrb-dim cells that had started to differentiate exhibited notably lower Plet1 staining (Fig. 3e). Under the same exposure settings, Plet1 was almost undetectable in 1 day-differentiated TSCs by immunostaining (Fig. 3e) and also significantly reduced by flow cytometry (Supplementary Fig. S3b). Strong immunostaining was again obvious in TGCs, easily identifiable by their characteristically large cell and nuclear size, both *in vitro* and in E8.5 trophoblast *in vivo* (Fig. 3f,g). Interestingly, flow cytometry analysis of 3-day differentiated TSCs, i.e. the time point when *Plet1* expression starts to become re-established, revealed that its up-regulation is confined to a subset of trophoblast cells, possibly those committed towards differentiation into TGCs (Supplementary Fig. S3b; Fig. 4). Overall, these biphasic expression dynamics upon TSC differentiation corresponded well with those observed during *in vivo* development.

As far as subcellular localisation is concerned, GPI anchored proteins such as the long, predominant Plet1 isoform are generally located on the extracellular face of the plasma membrane. They typically lack both a transmembrane domain and a cytoplasmic tail. Alternatively, they may be oriented towards the lumen within intracellular compartments^24^. Immunostaining of TSCs, without permeabilisation, revealed that Plet1 was indeed present on the cell surface where it co-localised with the placental cadherin Cdh3 (Fig. 3h), thus corroborating our flow cytometry results (Fig. 1e; Supplementary Fig. S1b). This membrane-specific staining was even more prominent when TSCs were grown on Matrigel (Fig. 3h). Permeabilisation even when performed post-Plet1 staining, necessary for example to detect the nuclear Esrrb, resulted in a more speckled Plet1 staining pattern, indicating that its membrane-association is relatively weak and sensitive to detergents, as would be expected from GPI-anchored proteins (Fig. 3e; Supplementary Fig. S3c).

Overall, these data revealed that Plet1 is a biphasically expressed and predominantly cell surface-localised protein present on TSCs and at even higher levels on differentiating TGCs but that is down-regulated in the intervening diploid trophoblast population.

### Overexpression of Plet1 promotes TSC differentiation

To gain more detailed insights into the physiological role of Plet1 in trophoblast, we performed both gain- and loss-of-function experiments. To this end, we first tested the effects of overexpressing the long and short isoforms of *Plet1* on TSC differentiation in transient transfection experiments (Fig. 4). We achieved good overexpression for the long isoform, and even dramatically higher levels for the short isoform due to its very low endogenous level of expression (Fig. 4c; Supplementary Fig. S4a). Overall, the most pronounced effects were observed for the (normally predominant) long, GPI-anchored Plet1 isoform, which accelerated the rate of TSC differentiation towards an intermediate EPC-like trophoblast and early TGCs, characterised by up-regulation of *Ets2*, *Gata3*, *Hand1* and *Prl8a9* (Fig. 4d). At even higher levels of overexpression (20–30x), a pronounced increase in expression of TGC markers such as *Plf* (*Prl2c2*), *Pl1* (*Prl3d1*), *Pl2* (*Prl3b1*), *Prl8a9* and *Ctsq* was observed (Supplementary Fig. S4a). The promotion of TGC differentiation, together with the absence of tightly packed, expanding TSC colonies, was also evident by the morphology of transfected cells (Supplementary Fig. S4b). However, the alternative major trophoblast differentiation route towards SynT (Fig. 4a) was markedly suppressed as indicated by the reduced levels of *Gcm1* and *Syna* expression (Fig. 4d). The effects of the short Plet1_002 isoform were generally similar although overall more modest despite its pronounced levels of overexpression, demonstrating that the main functions of Plet1 are conferred by its membrane-bound form. Thus, elevated *Plet1* expression levels drive TSCs out of their stem cell state and skew the ensuing differentiation trajectories towards the TGC pathway at the expense of SynT.

### Effect of Plet1 depletion

**Figure 5:**
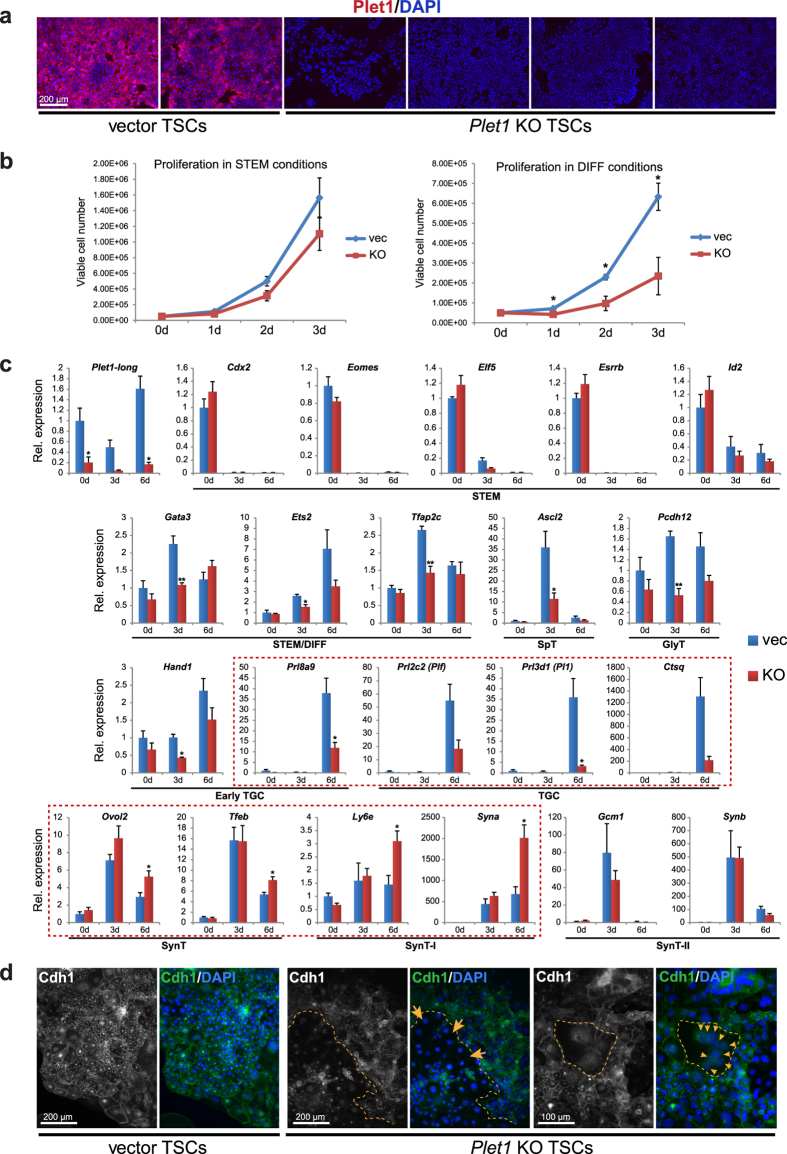
*Plet1* ablation by CRISPR/Cas9-mediated gene knockout promotes TSC differentiation into syncytiotrophoblast. (**a**) Immunofluorescence staining (without permeabilisation) of vector and *Plet1* KO TSCs, showing high levels of Plet1 (red) only in the vector control TSCs. Displayed are two independent vector clones and four independent *Plet1* KO clones (generated using three different gRNAs). Images were captured at exactly the same exposure settings; nuclear counterstain: DAPI. (**b**) Quantification of the proliferation rates of *Plet1* KO TSCs. In stem cell conditions (STEM; left), no significant differences were observed; however in differentiation conditions (DIFF; right), Plet1-null TSCs displayed a striking reduction in proliferation compared with vector controls. Data are mean of four biological replicates ±S.E.M. (*P < 0.05). (**c**) RT-qPCR analysis to determine the effect of *Plet1* depletion on stem cell maintenance and trophoblast differentiation (induced by removal of Fgf/CM). Loss of Plet1 has no significant effect on key stem cell markers; however, *Plet1*^−/−^ TSCs exhibit a notable failure to up-regulate markers of intermediate trophoblast (STEM/DIFF), spongiotrophoblast (SpT), glycogen trophoblast (GlyT), and trophoblast giant cells (TGCs). Concomitant with this under-representation of the TGC trajectory is an increased differentiation towards the syncytiotrophoblast (SynT) lineage, specifically layer I (SynT-I), as highlighted by the red boxes. Expression was normalised to *Sdha, Tbp*, and *Hprt1*. Data are mean of four biological replicates ±S.E.M. (*P < 0.05, **P < 0.005). (**d**) Immunofluorescence staining of 6-day differentiated vector control and *Plet1*^−/−^ TSCs for E-Cadherin (Cdh1) identifies a highly disorganised epithelial morphology and the frequent occurrence of multinucleated cells indicative of SynT (highlighted by dotted lines, arrows) in *Plet1*-deficient trophoblast cell cultures. Data are representative of 4 independent vector control and *Plet1* KO clones.

To complement the overexpression experiments, we aimed at assessing the effects of Plet1 depletion on TSCs. To this end, we performed CRISPR/Cas9-mediated gene knockout (KO) in TSCs, thereby demonstrating that this genome editing technology is feasible in this stem cell type. We designed and tested the efficiency of 3 different guideRNAs (gRNAs) targeting exons 1 and 2 (Supplementary Fig. S5a). To establish *Plet1* KO TSC lines, TSCs were transfected with the appropriate guideRNA-Cas9-EGFP constructs and single cell-sorted selecting EGFP-positive but Plet1-negative cells 2 days after transfection (Supplementary Fig. S5b). Clones arising from these single cells were tested for *Plet1* gene mutations by immunostaining and RT-qPCR, and the generation of functional null alleles was confirmed by sequencing (Fig. 5a; Supplementary Fig. S5c). A total of 4 empty vector control and 4 *Plet1* KO clones, derived from 3 different guideRNAs, were chosen for further analysis.

Although *Plet1* KO TSCs could be maintained in standard TSC culture conditions, they proliferated at a slower rate, an effect that was particularly obvious upon culture in differentiation medium (DIFF; Fig. 5b). This finding was generally in line with a largely unchanged gene expression programme in TSC conditions (“0d”, Fig. 5c). Assessing marker gene expression over a 6-day differentiation time course, however, revealed that *Plet1* KO TSCs exhibited a significant bias in differentiation into the major trophoblast subtypes. Thus, the up-regulation of TGC markers including *Prl8a9*, *Plf* (*Prl2c2*), *Pl1* (*Prl3d1*) and *Ctsq* was reduced, while markers of SynT (*Ovol2*, *Tfeb*), and specifically of the SynT-I layer (*Ly6e*, *Syna*), were increased compared to controls (Fig. 5c). The prevalence of syncytial cells upon differentiation was also evident as a striking phenotype when Cdh1 immunostaining was performed on *Plet1* KO TSCs (Fig. 5d; Supplementary Fig. S5d). This staining revealed an obvious loss of epithelial integrity with significantly disorganised Cdh1 localisation at the cell surface, and the appearance of large clusters of nuclei within a shared cell membrane.

Overall, these loss-of-function data strongly corroborated the results from the overexpression experiments, demonstrating that Plet1 levels bias the trajectories of TSC differentiation: high Plet1 levels favour differentiation towards EPC-like trophoblast leading into the TGC lineage, whereas lack of Plet1 preferentially induces SynT formation.

### Hypoxia can compensate for Plet1 deficiency

**Figure 6:**
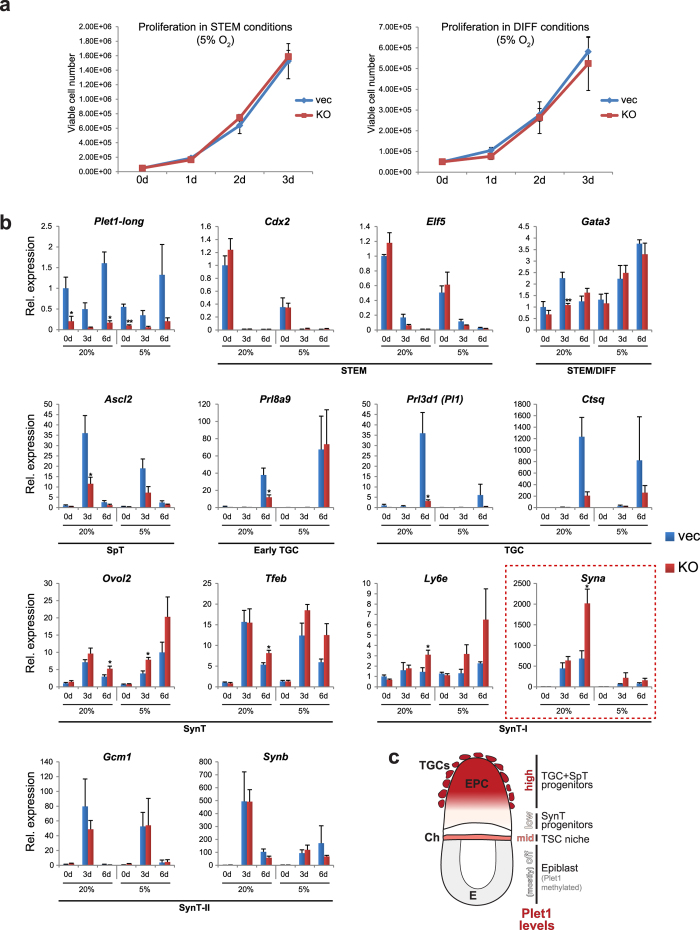
Hypoxia can compensate for Plet1 deficiency. (**a**) Quantification of the proliferation rates of *Plet1* KO TSCs cultured in hypoxic conditions (5% O_2_) revealed that *Plet1* KO TSCs no longer exhibited a proliferation defect, relative to vector controls, in either stem cell (STEM; left) or differentiation conditions (DIFF; right). (**b**) RT-qPCR analysis of the effect of *Plet1* depletion on TSC maintenance and differentiation (induced by removal of Fgf/CM) in hypoxic (5% O_2_) compared with ambient oxygen levels (~20% O_2_; data from Fig. 5c. As in 20% O_2,_ loss of Plet1 has no significant effect on prominent stem cell markers in hypoxic conditions; however the striking phenotype of increased syncytial trophoblast differentiation observed in 20% O_2_ is much reduced, exemplified by the failure to up-regulate *Syna*, a key marker of syncytiotrophoblast layer I (SynT-I). Expression data were normalised to *Sdha, Tbp*, and *Hprt1*, and are mean of four biological replicates ±S.E.M. (*P < 0.05, **P < 0.005). (**c**) Model of how the dynamic expression levels of Plet1 contribute to patterning the trophoblast compartment. Intermediate (“mid”) amounts of Plet1 are required to establish and maintain the TSC niche, made up *in vivo* by the distal extraembryonic ectoderm (i.e. close to the epiblast) and then the chorionic ectoderm (Ch) layer (highlighted in pink). Low or absent Plet1 in trophoblast cells at the base of the ectoplacental cone (lightest pink) favours differentiation towards syncytiotrophoblast (SynT) thereby establishing the placental labyrinth progenitor compartment. High Plet1 levels towards the tip and outer margins of the ectoplacental cone (dark red) drive differentiation along the trophoblast giant cell trajectory (including into trophoblast giant cells (TGCs), glycogen trophoblast cells and spongiotrophoblast (SpT)).

Oxygen tension is known to regulate cell fate decisions in the placenta. In particular, a low-oxygen environment has been reported to skew differentiation away from the SynT pathway^25^,^26^. We therefore aimed to test whether these opposing effects of hypoxia could suppress the Plet1-null phenotype. For this purpose, we cultured the same 4 vector control and *Plet1* KO TSC clones under 5% oxygen in stem cell or differentiation conditions over a period of up to 6 days. Examination of growth rates revealed that the proliferation defect of *Plet1* KO cells under ambient air (Fig. 5b) was abolished under hypoxic conditions (Fig. 6a). Furthermore, assessment of the panel of trophoblast differentiation markers showed that at least some effects of the *Plet1* KO phenotype were dampened (Fig. 6b): As previously reported, hypoxic conditions repressed differentiation towards the SynT lineage in vector control cells, as evidenced by the failure to up-regulate *Syna* and *Synb*. Similarly, *Plet1* KO cells – which in 20% O_2_ conditions exhibited an increased rate of SynT differentiation – also failed to up-regulate *Syna*, a key marker of SynT-I differentiation, in hypoxic conditions. Interestingly, the expression of another marker of SynT-I, *Ly6e*, was not repressed by culture in hypoxic conditions. This opens up the possibility that either, *Ly6e* marks an early precursor SynT-I population and that differentiation into this lineage is initiated but does not progress towards terminal differentiation; or that *Ly6e* exhibits a broader expression profile than *Syna*. The latter possibility is supported by expression of *Ly6e* in proliferating TSCs in which *Syna* is not expressed. Taken together, these data show that hypoxia was able to rescue both the proliferation defect as well as the differentiation bias of Plet1-deficient TSCs towards SynT.

## Discussion

Plet1 has emerged in several studies as a factor expressed in very restricted subpopulations of cells, yet the molecular function of Plet1 has remained largely elusive. Notably, many of these expression sites are epithelial in nature and exhibit stem or progenitor cell characteristics. For example, Plet1 has been detected in uterine luminal epithelium^27^ and in mammary gland epithelium^20^; furthermore, it demarcates very distinct populations of progenitor cells in hair follicles^11^,^28^, in pancreatic duct epithelium^16^, and in the thymus^16^,^17^,^29^. In the latter, Plet1-positive cells are capable of reconstituting the entire thymic epithelial microenvironment, thus demonstrating their considerable developmental plasticity. During embryonic development, the first site of *Plet1* expression is in the extraembryonic trophoblast compartment in few cells of the ExE immediately overlying the epiblast and in the EPC at E6.5. One day later, expression in the TSC progenitor niche – now constituted by the chorionic ectoderm – is still maintained; *Plet1* also remains expressed at very high levels in the EPC and in TGCs emerging at its margins^10^. Apart from this compelling expression pattern during early development, our interest in Plet1 arose from a genome-wide screen in which we had identified this gene as differentially methylated between ESCs and TSCs^30^ in a pattern shared only by very few other loci including the transcription factor Elf5^9^. These findings prompted the current investigation into the roles of Plet1 in early stem cell restriction and TSC biology.

Our data confirm that *Plet1* is one of an exquisitely small group of loci that is methylated and repressed in ESCs but hypomethylated and expressed in TSCs. This pattern is shared with *Elf5*, a transcription factor that we had previously identified as an epigenetically regulated lineage gatekeeper critical for the reinforcement of early cell fate restrictions^5^. Unlike Elf5, however, Plet1 is not capable of inducing TS-like fate in wild-type ESCs when overexpressed^9^. Yet, as we show here, the up-regulation of *Plet1* is necessary to activate key trophoblast genes in the *Dnmt1*-deficient ESC model. Thus, when *Plet1* is constitutively knocked down such that it remains suppressed upon shift of *Dnmt1*^−/−^ ESCs to TSC medium, the up-regulation of a number of markers characteristic of trophoblast such as *Cdx2*, *Elf5* and *Hand1* is abrogated. Interestingly, other genes such as *Tead4*, *Eomes*, and *Ascl2* are not or much less affected, pointing to a function of Plet1 in specific gene activation pathways. Given the cell membrane localisation of Plet1, this finding may suggest its involvement in the signal transduction cascades triggered by Fgf4 and/or CM. If and how Plet1 may modulate these pathways awaits further detailed experimental investigations. Our data reveal, however, that Plet1 is an essential factor to confer expression of a set of critical trophoblast genes, and is subject to an equally tight epigenetic regulation as the transcription factor Elf5.

Our further efforts were focused on elucidating the role of Plet1 in the trophoblast compartment where it is endogenously expressed. *Plet1* is present in TSCs but then abruptly down-regulated on day 1 of differentiation, indicating that its expression may be regulated by Fgf and/or CM. This is indeed corroborated by the sensitivity of *Plet1* levels to Mek inhibition^23^, suggesting that *Plet1* expression is under the control of Fgf->Erk signalling. During further differentiation, *Plet1* is again up-regulated to levels that even exceed those observed in TSCs. How this second wave of expression is regulated is still unknown, but it is interesting to note that this distribution is shared by few other factors such as *Gata3* and, intriguingly, also by phosphorylated Erk^31^,^32^.

Our overexpression experiments reveal that excessive amounts of Plet1 promote TSC differentiation, predominantly into the TGC trajectory combined with a significant depletion of SynT. This effect is in line with the very high Plet1 levels detected in EPC and in TGCs emerging at its margins, and suggests that Plet1 is not only associated with these cells but may in fact be causative of triggering their differentiation. Leading on from this, the separation of 3-day differentiated TSCs into Plet1-low and -high cells may indeed reflect their commitment towards a SynT and TGC differentiation route, respectively.

In perfect agreement with the overexpression experiments, we observe the opposite phenotype in TSCs in which *Plet1* was genetically ablated by CRISPR-Cas9-mediated mutagenesis. These cells exhibit a preference towards differentiation into SynT at the expense of the TGC lineage, an effect that is under the regulation of oxygen tension. The similarity in phenotype between *Plet1* KO TSCs and TSCs lacking the aryl hydrocarbon receptor nuclear translocator *Arnt*, a key component of the hypoxia-inducible factor (Hif), is intriguing^25^,^33^, in particular since hypoxia can at least partially rescue the SynT differentiation bias of *Plet1* null TSCs. Thus it is tempting to speculate that a crosstalk between Plet1 and Hif, independent of oxygen tension, is important for normal trophoblast differentiation.

Overall, these data highlight that Plet1 is an important modulator of the major TSC differentiation pathways. Trophoblast cells exhibiting high Plet1 levels are prone to differentiate towards TGCs (and/or spongiotrophoblast) while cells with low/absent Plet1 preferentially form SynT. Combined with its presence on the cell surface this makes Plet1 an important and convenient tool to sort trophoblast cell populations downstream of TSC identity into TGC- and SynT-lineage precursors.

From a physiological point of view, our findings further show that the kinetics of Plet1 expression may in fact be instrumental for the various trophoblast subpopulations to emerge. Plet1 exhibits an unusual expression pattern as it is present in the ExE and chorion (the TSC progenitor niche) as well as in the proximal regions of the EPC, but absent from the intervening layer of diploid trophoblast cells at the base of the EPC. We show that this biphasic expression pattern is recapitulated *in vitro* in TSC differentiation time course experiments. Numerous marker gene analyses have demonstrated that the base of the EPC, i.e. the Plet1-depleted region, harbours the population of cells that will go on to fuse into SynT and form the developing placental labyrinth^34^,^35^,^36^,^37^,^38^. Thus, not only does the modulation of Plet1 levels introduce a differentiation bias in TSCs *in vitro*, but it establishes essential patterning cues of the trophoblast compartment *in vivo* that ultimately enables the differentiation of the major trophoblast subtypes in the correct spatial position (Fig. 6c).

Taken together, we here provide novel insights into the role of the cell surface protein Plet1 in TSC maintenance and its involvement in directing trophoblast cell fates.

## Methods

### Stem cell culture

Wild-type ESC lines were ES-E14 (originally termed E14tg2a) and ES-J1 (obtained from the Mutant Mouse Regional Resource Center (MMRRC) and a kind gift of the Masutani laboratory, respectively). ESC line (*Dnmt1*^−/−^) used for shRNA transfections was originally derived from 129Sv × (*M. cast* × 129Sv) blastocysts, and carries a homozygous mutation (*Dnmt1*^*s/s*^) in the gene encoding DNA methyltransferase 1 (ref. 39). Wild-type TSC lines were blastocyst-derived TS-Rs26 (used throughout unless indicated) and TS-EGFP (both a kind gift of the Rossant lab, Toronto, Canada). ESCs and TSCs were cultured in routine conditions as described previously^9^,^30^. Briefly, ESCs were grown on gelatin-coated tissue culture dishes, with or without mouse embryonic fibroblast (MEF) cell layers, in standard ESC medium: 15% foetal bovine serum (FBS), 1 mM sodium pyruvate (ThermoFisher Scientific 11360-039), 1X Anti-mycotic/Antibiotic (ThermoFisher Scientific 15240-062), 50 μM 2-mercaptoethanol, 1X non-essential amino acids (ThermoFisher Scientific 11140-035), 10 ng/ml Lif (Cambridge Stem Cell Institute) in DMEM with L-Glutamine (ThermoFisher Scientific 41965-039). Medium was changed every 1–2 days, and cells passaged when plenty of colonies were visible. TSCs were cultured in standard TSC conditions: 20% foetal bovine serum (FBS), 1 mM sodium pyruvate (ThermoFisher Scientific 11360-039), 1X Anti-mycotic/Antibiotic (ThermoFisher Scientific 15240-062), 50 μM 2-mercaptoethanol, 50 ng/ml bFGF (Cambridge Stem Cell Institute), and 1 μg/ml heparin in RPMI 1640 with L-Glutamine (ThermoFisher Scientific 21875-034), with 70% of the medium pre-conditioned on MEFs (CM). The medium was changed every two days, and cells passaged before reaching confluency. Trypsinisation (0.25% Trypsin/EDTA) was carried out at 37 °C for about 5 min. Differentiation medium consisted of unconditioned TSC medium without bFGF and heparin. For hypoxia experiments, cells were cultured in an O_2_/CO_2_ incubator (Sanyo/Panasonic MCO-19M) at 5% O_2_, 5% CO_2_, 90% N_2_ for the number of days indicated.

### Constructs and Transfections

For *Plet1* isoform overexpression, both open reading frames were amplified and cloned from TSC cDNA, sequence-verified and used to generate pCAG-Plet1_001-IRES-EGFP (longer GPI-anchored isoform) and pCAG-Plet1_002-IRES-EGFP (short isoform). These constructs were used, in co-transfection with pCAG-MCS-V5-Puro, to generate TS-Rs26 cells with transient *Plet1* overexpression that were selected with Puromycin. For *Plet1* knockdown, two shRNA constructs were designed as previously described^40^. Specifically, shRNAs were cloned between the 5′ and 3′ flanking sequences derived from an endogenous microRNA primary transcript (miR-30). ShRNA sequences were obtained from RNAi Codex (http://codex.cshl.edu/): shRNA1 (HP_294738) targeting both *Plet1* isoforms and shRNA2 (HP_294420) specific to the longer GPI-anchored isoform, (Plet1-001). Each strand was ordered as single-stranded oligonucleotides that were annealed *in vitro*, cloned into the pQuiet vector (kindly donated by C. Krueger, Reik lab) and sequence-verified through restriction digest and sequencing. For CRISPR/Cas9 mediated knockout of *Plet1*, gRNAs were designed using the CRISPR.mit.edu design software or selected from previously published genome-wide CRISPR screening studies^41^. All gRNA sequences were subjected to nucleotide BLAST searches and only those with high specificity were selected to limit the potential for off-target effects. As above, gRNA sequences were ordered as single-stranded oligonucleotides, annealed *in vitro*, and used to generate gRNA + Cas9.2A.EGFP single plasmid constructs (Plasmid #48138 Addgene). After sequence verification, empty vector Cas9.2A.EGFP construct and *Plet1*-specific gRNA + Cas9.2A.EGFP constructs were used to generate Cas9 vector TSCs and *Plet1* KO TSCs, respectively, as described (Supplementary Fig. S3). All transfection experiments were carried out with Lipofectamine 2000 (ThermoFisher Scientific 11668019) reagent according to the manufacturer’s protocol.

### DNA methylation analysis

Genomic DNA was extracted from wild-type ESCs (ES-E14 & ES-J1) and TSCs (TS-Rs26 & TS-EGFP) & *Dnmt1*^−/−^ ESCs by lysis in 100 mM Tris-HCl pH 8.5, 5 mM EDTA pH 8.0, 0.2% SDS, 200 mM NaCl, 200 μg/ml Proteinase K at 60 °C overnight and the DNA purified by phenol-chloroform extraction, followed by 2-propanol/75% ethanol purification, and subsequently dissolved in water overnight.

One to two micrograms of DNA were used for bisulphite conversion using the EpiTect kit (Qiagen 59104), according to the manufacturer’s instructions. Genomic regions of interest were amplified in simple or nested PCRs (bisulphite DNA-specific primer pairs provided (Supplementary Table S1), products cloned into pGEM-T Easy Vector system (Promega) and 6–9 colonies per sample picked, and plasmids extracted (QIAprep Spin Miniprep Kit, Qiagen) and sequenced. Non-clonality of sequenced alleles was confirmed.

### RT-qPCR expression analysis

Total RNA was extracted using TRI reagent (Sigma T9424) followed by DNase treatment using the TURBO-DNA-free kit (Life Technologies AM1907) according to the manufacturers’ instructions. One to two micrograms of total RNA (when sufficient material was available) were used for cDNA synthesis with RevertAid H-Minus reverse transcriptase (Thermo Scientific EP0451), according to manufacturer’s instructions, using random hexamer primers (Thermo Scientific SO142) and RiboLock RNase inhibitor (Thermo Scientific EO0381). A reaction without reverse transcriptase (RT-) was carried out to control for DNA contamination.

Quantitative PCR was performed using SYBR Green Jump Start Taq Ready Mix (Sigma S4438) and intron-spanning primer pairs (Supplementary Table S1) on a Bio-Rad CFX96 or CFX384 thermocycler. For the analysis of *Plet1* expression, where not further specified primers common to both predicted isoforms were used (Plet1-F/R). The reaction protocol included an initial denaturation step at 94 °C for 2 min in order to activate the JumpStart Taq, followed by 40 cycles of denaturation (94 °C), annealing (60–65 °C; primer-dependent), and extension (72 °C). A melting curve was performed to verify the presence of a single PCR product.

Cycle threshold (Ct) values were analysed using Microsoft Excel software, according to the comparative Ct method (ΔΔCt). The Ct values of three technical replicates were examined to ensure a discrepancy of less than 0.5 cycles. Ct values were normalised to housekeeping genes as specified, such as *Sdha*, *Hprt1*, *Gapdh*, *Dynein, Tbp, Pgk1*. The level of gene expression was displayed as mean (relative to the control sample); error bars indicate standard error of the means (S.E.M.) of at least three replicates. Where appropriate, student’s t-tests or ANOVA was performed to calculate statistical significance of expression differences (p < 0.05).

### Immunofluorescence staining

For staining of cultured cells, cells were grown on coverslips or in droplets of Matrigel (BD Biosciences 356231), fixed with 4% paraformaldehyde for 10 min and permeabilised with PBS, 0.1% Triton X-100 for 30 min (the permeabilisation step was omitted where specified). Staining of paraffin sections of E8.5 mouse conceptuses was performed as previously described^42^. Blocking was carried out by incubating with PBS, 0.1%Tween-20, 0.5%BSA (PBT/BSA) for 15 min, prior to antibody staining. For double immunofluorescence staining of Plet1 and Esrrb, cells were fixed, blocked, stained without permeabilisation for Plet1, post-fixed for 10 min in 4% paraformaldehyde, then permeabilised and stained for Esrrb. Primary antibodies and dilutions (in PBT/BSA) used were rat anti-Plet1 1:300 (Nordic MUbio MUB1512P) and rat anti-Plet1 1:75 (a kind gift from C. Blackburn), mouse anti-Esrrb 1:400 (R&D Systems MAB1499), anti-Tfap2c 1:400 (R&D Systems AF5059), mouse anti-Cdh3 1:100 (NeoMarkers MS-1741-S0), and mouse anti-Cdh1 1:100 (BD Biosciences 610181). Primary antibodies were detected with the appropriate secondary AlexaFluor 488 or 568 (ThermoFisher Scientific) antibodies. Nuclei were counter-stained with 4,6-diamidino-2-phenylindole (DAPI), and coverslips were mounted on glass microscope slides in 50% glycerol. Photographs were taken with an Olympus BX41 or BX61 epifluorescence microscope or a Zeiss LSM 780 confocal microscope. Images were analysed with Adobe Photoshop CS6.

### Flow cytometry

Wild-type ES-E14, *Dnmt1*^−/−^ ESCs and TS-Rs26 cells were stained for Plet1, diluted 1:100–1:400 (Nordic MUbio MUB1512P), to assess differential protein levels. Similarly, TS-Rs26 cells transfected with *Plet1*-targeting CRISPR/Cas9 constructs were stained for Plet1 in order to enrich for the knockout population. Initially, cells were trypsinised, washed, gently resuspended, filtered through a 40 μM cell strainer and resuspended in 2% foetal bovine serum in PBS (PBS, 2% FBS). Sequential stainings were performed at 4°C, by incubation with primary and secondary antibodies for 30 min each. Cells were resuspended in a final volume of 500 μl of PBS, 2% FBS (supplemented with 1 μg/ml DAPI to assess cell viability). Analysis (at least 20,000 live cells were measured for each sample/condition) and/or sorting was performed using a Becton Dickinson FACSAria or a Fortessa cell sorter. Raw datasets were analysed using FlowJo software and graphically displayed as dot plots or histograms.

### Proliferation assay

To quantify proliferation rates of Cas9 vector and *Plet1* KO TSCs, 50,000 cells were plated in either stem cell (+Fgf/CM) or differentiation (TS base media only) conditions and harvested every 24 hrs over a three day period. Cells were trypsinised, and a viable cell count was performed using the Muse Count & Viability Assay Kit (Merck Millipore MCH100102) and run on the Muse cell analyser (Merck Millipore), according to manufacturer’s instructions.

## Additional Information

**How to cite this article**: Murray, A. *et al*. Plet1 is an epigenetically regulated cell surface protein that provides essential cues to direct trophoblast stem cell differentiation. *Sci. Rep*. **6**, 25112; doi: 10.1038/srep25112 (2016).

## Supplementary Material

Supplementary Information
