# Supplementary material for: Plet1 is an epigenetically regulated cell surface protein that provides essential cues to direct trophoblast stem cell differentiation
Source: Sci Rep. 2016 Apr 28;6:25112. doi: 10.1038/srep25112 (PMC4848516; doi:10.1038/srep25112)
Supplement: Supplementary Information [file srep25112-s1.pdf]

Supplementary Information

Plet1 is an epigenetically regulated cell surface protein that provides essential cues to direct trophoblast stem cell differentiation

Alexander Murray[1,2], Arnold R. Sienerth[1] and Myriam Hemberger[1,2,*]

1 Epigenetics Programme, The Babraham Institute, Babraham Research Campus, Cambridge CB22 3AT, UK

2 Centre for Trophoblast Research, University of Cambridge, Downing Street, Cambridge CB2 3EG, UK

* corresponding author

Dr. Myriam Hemberger
Epigenetics Programme
The Babraham Institute
Babraham Research Campus
Cambridge CB22 3AT
United Kingdom
Tel: +44 (0)1223 496 534
Fax: +44 (0)1223 496 034
Email: myriam.hemberger@babraham.ac.uk

Running head: Plet1 levels modulate TSC fate

Keywords: Trophoblast stem cells, lineage gatekeeper, DNA methylation, epigenetics, self-renewal, control of differentiation

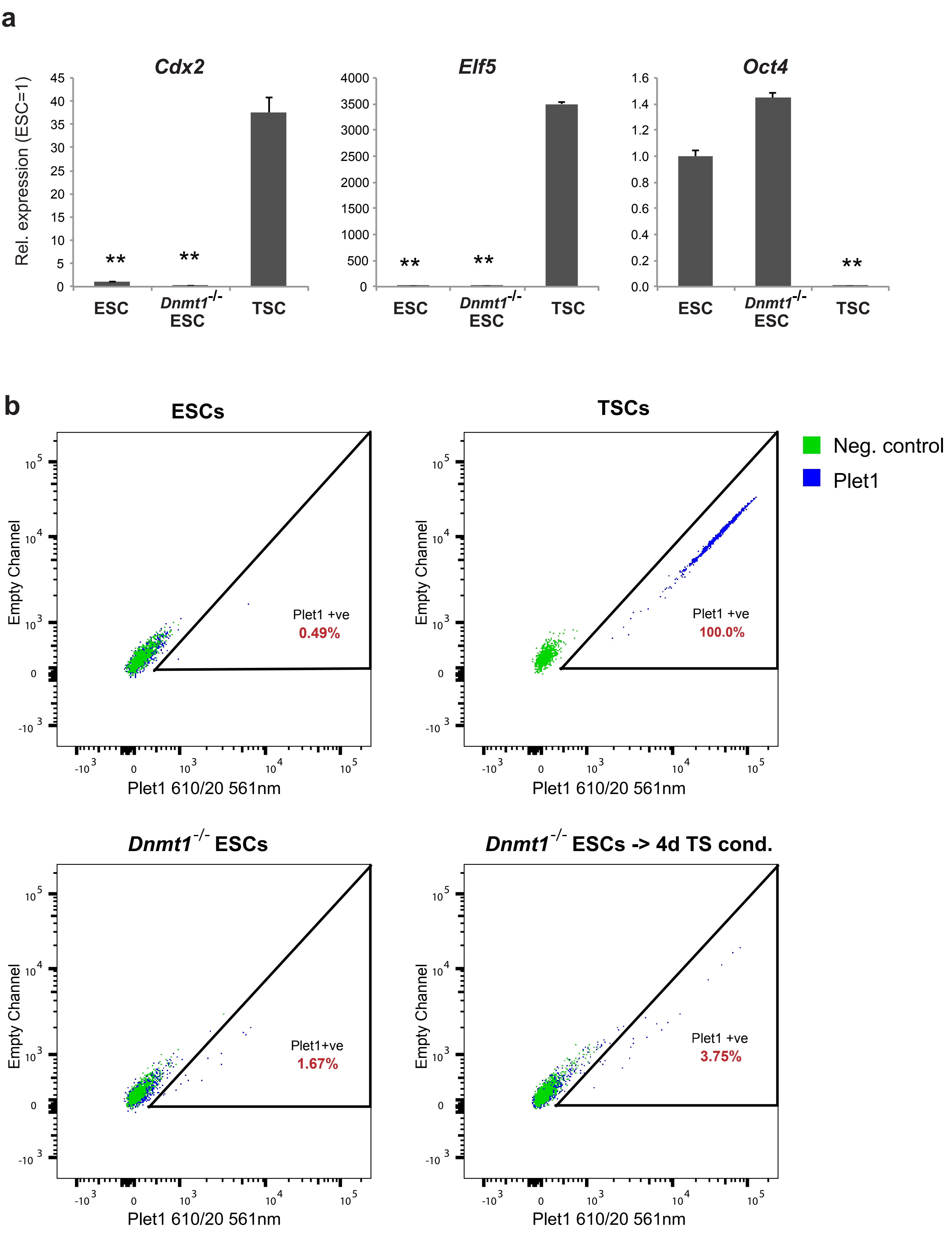
a
Cdx2
Elf5
Oct4
Rel. expression (ESC=1)
45
40
35
30
25
20
15
10
5
0
4000
3500
3000
2500
2000
1500
1000
500
0
1.6
1.4
1.2
1.0
0.8
0.6
0.4
0.2
0.0
**
ESC
Dnmt1-/- ESC
TSC
b
ESCs
TSCs
Dnmt1-/- ESCs
Dnmt1-/- ESCs -> 4d TS cond.
Neg. control
Plet1
Empty Channel
Plet1 610/20 561nm
10^5
10^4
10^3
0
-10^3
Plet1 +ve
0.49%
100.0%
Plet1+ve
1.67%
3.75%

Supplementary Figure S1. *Plet1* is a trophoblast-expressed cell surface protein. (a) Expression of lineage marker genes *Cdx2*, *Elf5* and *Oct4* (*Pou5f1*) in ESCs, $Dnmt1^{-/-}$ ESCs and TSCs as shown in Fig. 1c. Data are mean of three replicates displayed ± S.E.M. (**P < 0.005). **(b)** Flow cytometry analysis to identify Plet1-positive cells in $Dnmt1^{-/-}$ ESCs grown in ESC conditions or after 4 days of culture in TSC conditions. Wild-type ESCs and TSCs were used as control. The triangles demarcate the gating for Plet1-positive cells (blue pixels) against cell line-specific negative controls (green pixels); the percentage of Plet1-positive cells within the entire cell population is displayed for each group. For each sample, a total of 20,000 cells were analysed. As shown in Fig. 1e, TSCs are homogenously positive for Plet1 whereas ESCs are negative. Consistent with the RT-qPCR data (Fig. 1c), there is only a marginal increase in Plet1-positive cells in $Dnmt1^{-/-}$ ESCs; however, this fraction increases as cells undergo trans-differentiation towards trophoblast-like cells upon culture in TSC conditions, confirming again that the *Plet1* mRNA amounts determined by RT-qPCR (Fig. 2b) and the Plet1 surface protein levels are closely correlated.

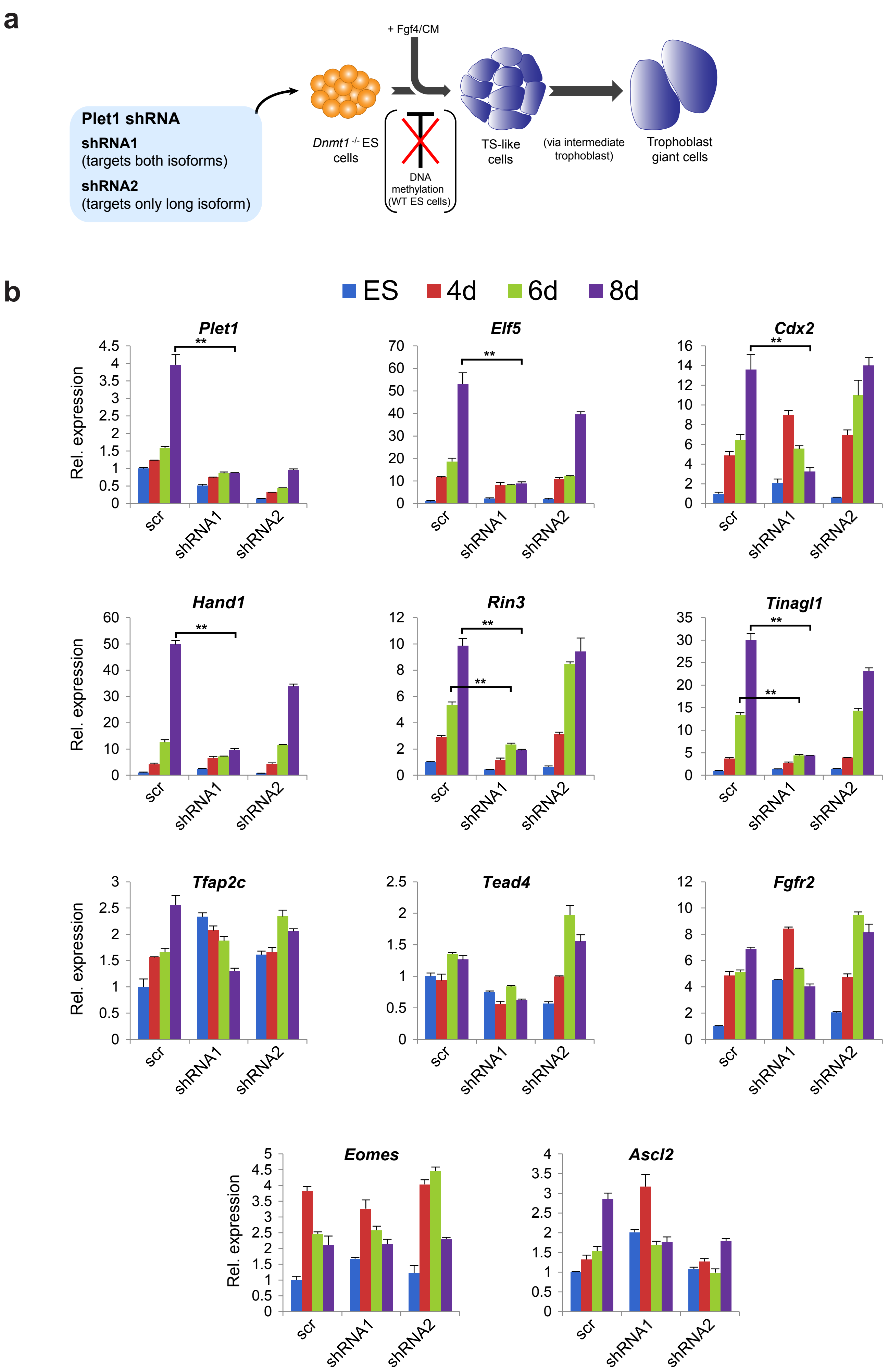
a
+ Fgf4/CM
Plet1 shRNA
shRNA1
(targets both isoforms)
shRNA2
(targets only long isoform)
Dnmt1 -/- ES cells
DNA methylation (WT ES cells)
TS-like cells
(via intermediate trophoblast)
Trophoblast giant cells
b
ES
4d
6d
8d
Plet1
Elf5
Cdx2
Hand1
Rin3
Tinagl1
Tfap2c
Tead4
Fgfr2
Eomes
Ascl2
Rel. expression
scr
shRNA1
shRNA2
**

Supplementary Figure S2. *Plet1* is necessary to induce trophoblast trans-differentiation in ESCs. (a) *Dnmt1*$^{-/-}$ ESCs readily trans-differentiate into trophoblast cell types on transfer into TSC culture conditions (+Fgf/CM). **(b)** *Dnmt1*$^{-/-}$ ESCs were transfected with *Plet1* isoform-specific knockdown constructs: scrambled (scr), targeting both isoforms (shRNA1), or specific to the longer GPI-anchored isoform (shRNA2). *Dnmt1*$^{-/-}$ ESC clones with stable *Plet1* knockdown were established in ESC media (+Lif), and then induced to trans-differentiate into trophoblast by culture in TSC media alongside untransfected *Dnmt1*$^{-/-}$ ESCs (s/s). shRNA1-mediated *Plet1* knockdown prevents up-regulation of trophoblast markers, notably *Elf5*, *Cdx2* and *Hand1*, as well as *Rin3* and *Tinagl1* that were previously identified as trophoblast lineage signature genes[9], suggesting that *Plet1* is required for trophoblast trans-differentiation from ESCs. Data are mean of three replicates ± S.E.M., and representative of two experiments.

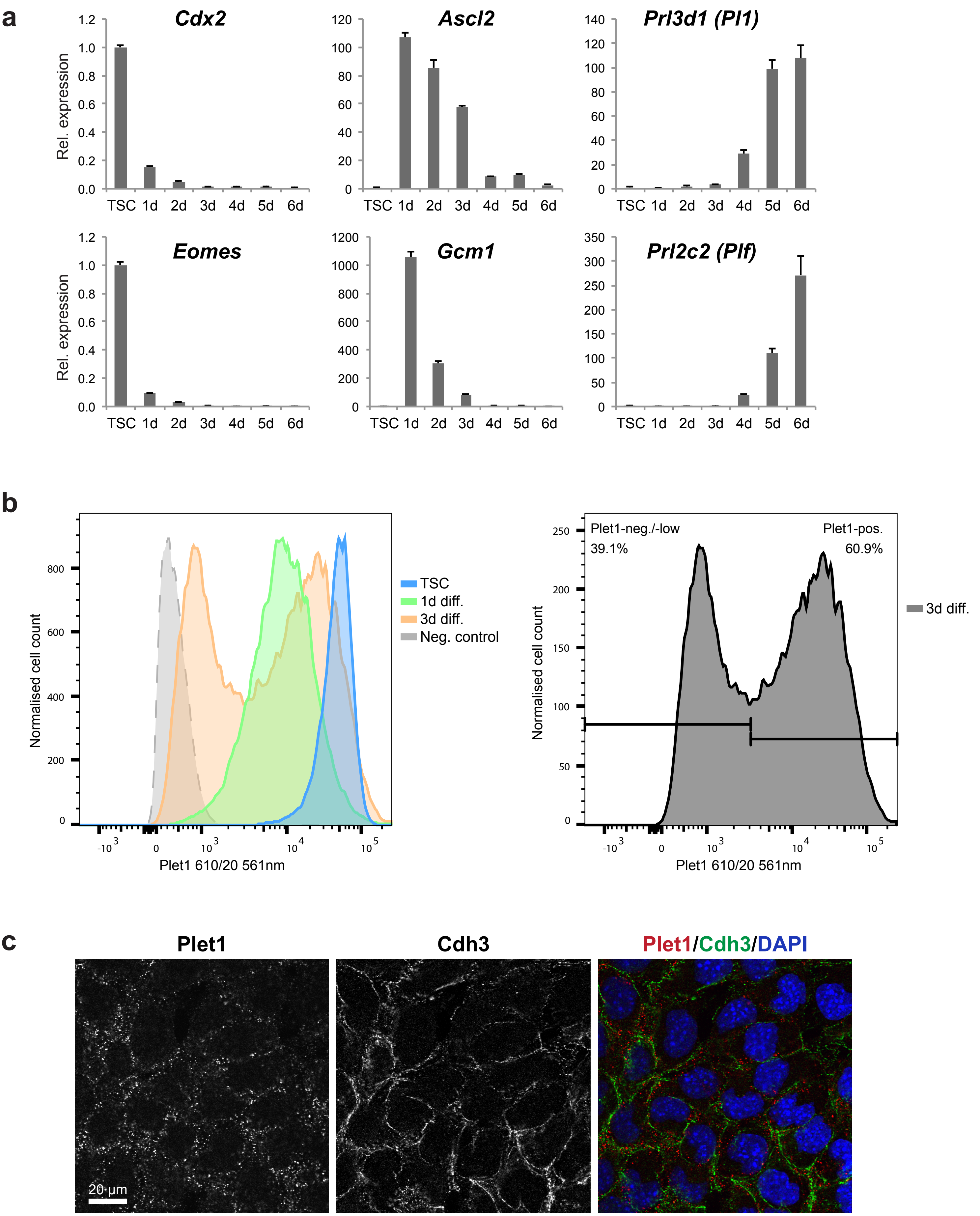
a
Cdx2
Ascl2
Prl3d1 (Pl1)
Eomes
Gcm1
Prl2c2 (Plf)
Rel. expression
TSC 1d 2d 3d 4d 5d 6d
b
Normalised cell count
Plet1 610/20 561nm
TSC
1d diff.
3d diff.
Neg. control
Plet1-neg./-low
39.1%
Plet1-pos.
60.9%
3d diff.
c
Plet1
Cdh3
Plet1/Cdh3/DAPI
20 µm

Supplementary Figure S3. Plet1 regulation with trophoblast differentiation.

(a) Relative expression levels of stem cell and differentiation marker genes during the 7-day time course of TSC differentiation as shown in Fig. 3c. Data represent the mean of six biological replicates ± S.E.M. **(b)** Flow cytometry analysis for Plet1 during TSC differentiation. For each sample, a total of 20,000 cells were analysed that are displayed as histogram of staining intensity (x-axis). Surface protein levels recapitulate the down-regulation of Plet1 observed on the mRNA level (Fig. 3c) between TSCs and ~1 day (20 hours)-differentiated TSCs. Upon further differentiation at day 3, the majority of cells re-acquire Plet1 similar to the results observed by RT-qPCR. Quantification of cell fractions that are either negative (or low) for Plet1 or positive for Plet1 at 3d of differentiation was carried out by appropriate gating as shown in the right-hand graph. **(c)** Confocal images of double-immunofluorescence staining for Plet1 and Cdh3 of TSCs with prior permeabilisation. Nuclear counterstain: DAPI.

Murray_Suppl. Figure S4

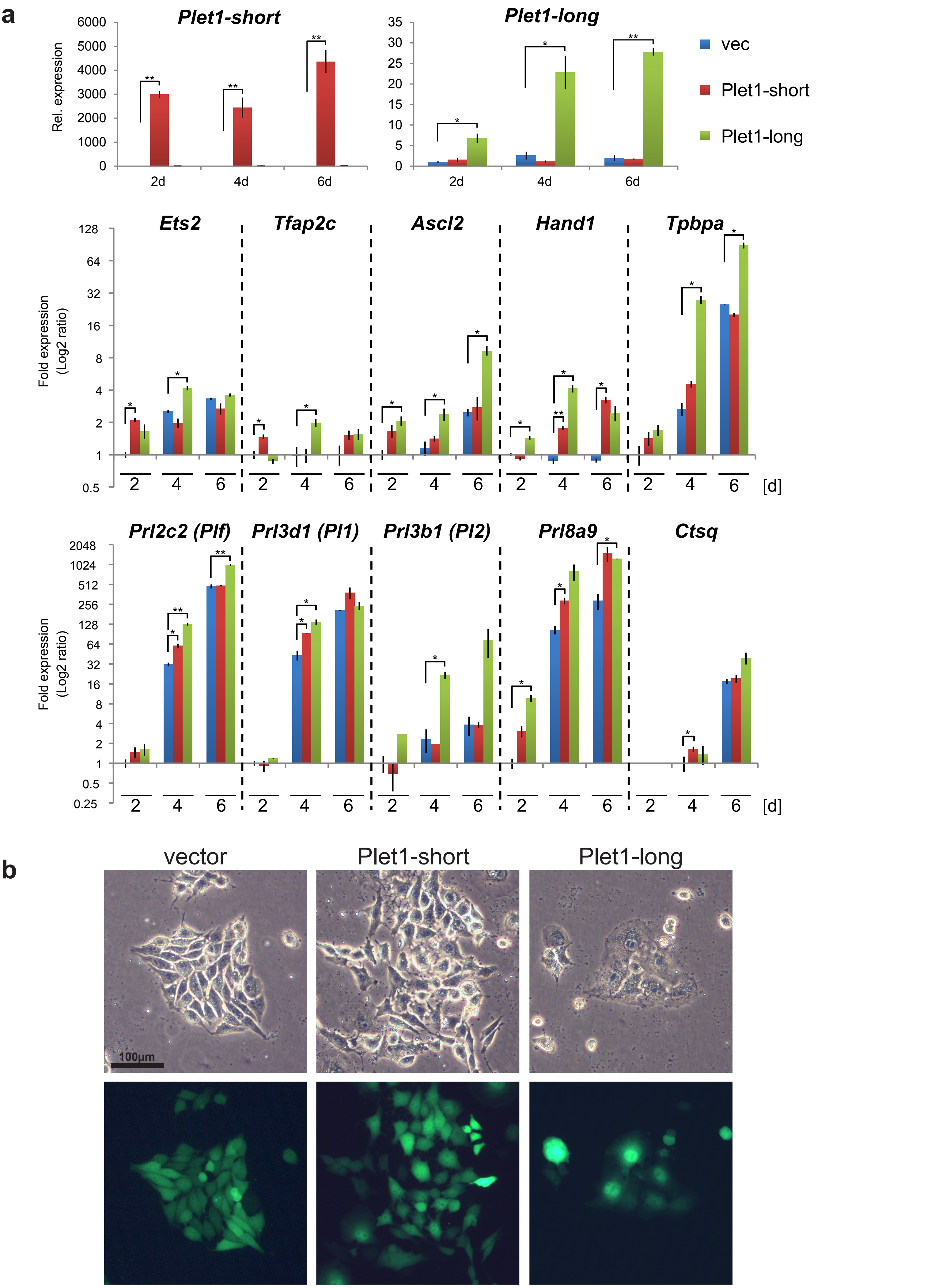

Supplementary Figure S4. *Plet1* overexpression promotes differentiation of TSCs. (a) Effect of high-level over-expression of *Plet1* on TSC differentiation. Wild-type TSCs were transfected with either the short isoform (Plet1-short) or the longer GPI-anchored isoform (Plet1-long). Displayed is the effect of *Plet1* isoform expression (upper) on markers of intermediate trophoblast (middle), and more differentiated giant cell markers (lower). Higher over-expression levels were achieved in this experimental series in particular for Plet1-long (Plet1_001) at 4 days, compare to Fig. 4c,d, resulting in more pronounced differentiation into the trophoblast giant cell lineage. Data are mean of three replicates ± S.E.M. (*P<0.05, **P<0.005). **(b)** Phase contrast (upper row) and fluorescent photomicrographs (lower row) of TSCs transfected with vector, Plet1-short or Plet1-long expression constructs. EGFP fluorescence is indicative of transfected cells as the vector contains an IRES-EGFP sequence. Note the more differentiated appearance of Plet1 over-expressing cells despite continued culture in TSC conditions, in particular for the Plet1-long isoform in which the vast majority of cells have differentiated into trophoblast giant cells obvious by their large cell and nuclear size. Photos were taken at identical exposure settings 4 days post-transfection.

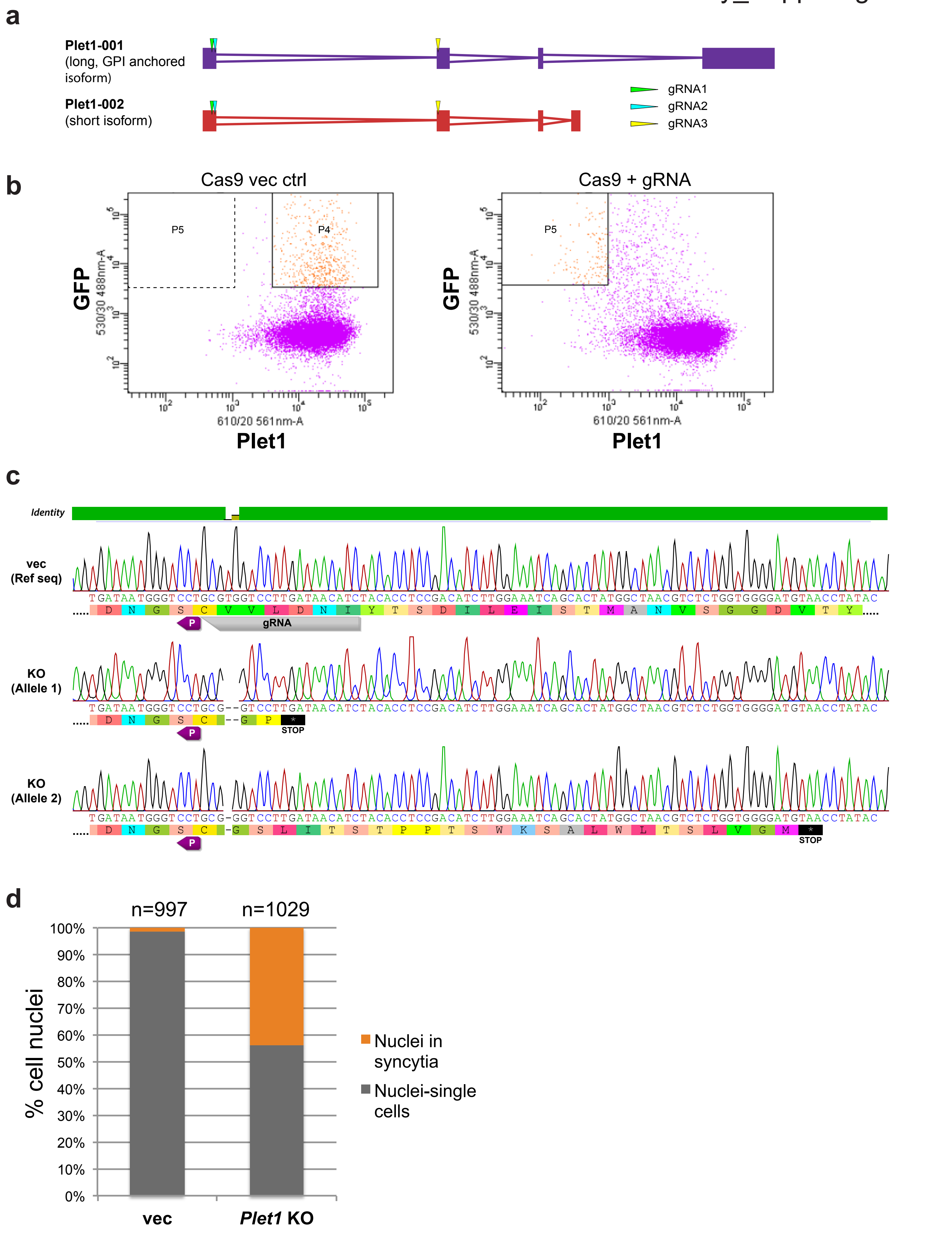
a
Plet1-001
(long, GPI anchored isoform)
Plet1-002
(short isoform)
gRNA1
gRNA2
gRNA3
b
Cas9 vec ctrl
Cas9 + gRNA
P5
P4
GFP
530/30 488nm-A
610/20 561nm-A
Plet1
c
Identity
vec
(Ref seq)
TGATAATGGGTCCTGCGTGGTCCTTGATAACATCTACACCTCCGACATCTTGGAAATCAGCACTATGGCTAACGTCTCTGGTGGGGATGTAACCTATAC
D N G S C V V L D N I Y T S D I L E I S T M A N V S G G D V T Y
P
gRNA
KO
(Allele 1)
TGATAATGGGTCCTGCG--GTCCTTGATAACATCTACACCTCCGACATCTTGGAAATCAGCACTATGGCTAACGTCTCTGGTGGGGATGTAACCTATAC
D N G S C --G P *
STOP
KO
(Allele 2)
TGATAATGGGTCCTGCG-GGTCCTTGATAACATCTACACCTCCGACATCTTGGAAATCAGCACTATGGCTAACGTCTCTGGTGGGGATGTAACCTATAC
D N G S C -G S L I T S T P P T S W K S A L W L T S L V G M *
STOP
d
n=997
n=1029
% cell nuclei
100%
90%
80%
70%
60%
50%
40%
30%
20%
10%
0%
vec
Plet1 KO
Nuclei in syncytia
Nuclei-single cells

Supplementary Figure S5. *Plet1* knockout strategy using CRISPR/Cas9.

(a) Schematic diagram displaying the two isoforms of *Plet1* and the location of gRNA binding sites. All gRNA sequences were subjected to nucleotide BLAST searches and only those with high specificity were selected to limit the potential for off-target effects. **(b)** Wild-type TSCs were transfected with either empty vector Cas9.2A.EGFP construct (left; Cas9 vec ctrl) or *Plet1*-specific gRNA + Cas9.2A.EGFP construct (right; Cas9 + gRNA) and maintained in TSC media for two days. Cells were then stained for Plet1 and sorted by flow cytometry. For the vector control, the successfully transfected GFP/Plet1 positive population (P4) was collected; whereas in the Cas9 + gRNA samples the GFP-positive but Plet1 negative population (P5), not present in the vector control, was enriched for. Desired populations were single-cell sorted into 96-well plates facilitating the derivation of clonal cell lines. **(c)** Schematic diagram (generated using Geneious sequence analysis software) showing sequence traces for Cas9 vector control and both alleles of a representative *Plet1* KO clone. Displayed is the target locus for gRNA1 (in exon 1 of *Plet1*). Note, indel mutations on both alleles cause frame-shift mutation and a premature stop codon (STOP). P = protospacer adjacent motif (PAM); gRNA = guide RNA. **(d)** Quantification of cell nuclei that appear as single cells or in syncytia in vector control (vec) and *Plet1*$^{-/-}$ TSCs after 6 days of differentiation. Quantification was performed on 3 independent clones each; the total number of nuclei counted is given.

Supplementary Table S1

Primer name	Sequence (5'-3')
Ascl2_F	AGCCCGATGGAGCAGGAG
Ascl2_R	CCGAGCAGAGGTCAGTCAGC
Cdh1_F	AGTTTACCCAGCCGGTCTTT
Cdh1_R	CCGGTGTCCCTATTGACAGT
Cdx2_F	AGTGAGCTGGCTGCCACACT
Cdx2_R	GCTGCTGCTGCTTCTTCTTGA
Ctsq_F	AATTGGCTATGGTTATGTGGGA
Ctsq_R	TCACACAGTAGGGTATTGGG
D930020E02(SynB)_F	TCCGGAAAGGGACCTGCCCA
D930020E02(SynB)_R	CAGCAGTAGTGCGGGGTGCC
Dynein_F	GACCTCAGGCTCAGACGAAGAC
Dynein_R	AAGACGCTCATGGCATCACA
Elf5_F	ATTCGCTCGCAAGGTTACTCC
Elf5_R	GGATGCCACAGTTCTCTTCAGG
Eomes_F	TCGCTGTGACGGCCTACCAA
Eomes_R	AGGGGAATCCGTGGGAGATGGA
Esrrb_F	AGTACAAGCGACGGCTGG
Esrrb_R	CCTAGTAGATTCGAGACGATCTTAGTCA
Ets2_F	GACCAAGTGGCCCCTGTCGC
Ets2_R	GGCCCGTGGGCACTTCTTGG
Fgfr2_F	TGCAGCTAGGACGGTAGACA
Fgfr2_R	GTCCAGTACGGTGCTCTCTG
Gapdh_F	ACATCTCACTCAAGATTGTCAGCA
Gapdh_R	ATGGCATGGACTGTGGTCAT
Gata3_F	AGGCAACCACGTCCCGTCCT
Gata3_R	CGGTGTGGTGGCTGCTCAGG
Gcm1_F	ACCCCTGAAGCTTATTCCCT
Gcm1_R	TCGCCTTTGGACTGGAAA
Gm52-(SynA)_F	CCTCACCTCCCAGGCCCCTC
Gm52-(SynA)_R	GGCAGGGAGTTTGCCCACGA
Hand1_F	GAACTCAAAAAGACGGATGGTGG
Hand1_R	CGCCCAGACTTGCTGAGG
Hprt1_F	GTCATGCCGACCCGCAGTCC
Hprt1_R	GGCCACAATGTGATGGCCTCC
Id2_F	ACTCGCATCCCACTATCGTC
Id2_R	AATTCAGATGCCTGCAAGGA
Id3_F	CAGCTGAGCTCACTCCGGAAC
Id3_R	CCAGAGTCCCAGGGTCCCAA
Ly6e_F	GATGTCTGCCACTTCCAACA
Ly6e_R	CATCAGGGAATGAACTTGCTC
Ovol2_F	AACTCCAGAGCTTCACGACG
Ovol2_R	GCATGTGCCGGTGGTAAACT
Pcdh12_F	GCTGCTTTTGCGGAACGGAA
Pcdh12_R	TTTGGGCTGGAATTGGCCCT
Pgk1_F	CTGACTTTGGACAAGCTGGACG

Pgk1_R	GCAGCCTTGATCCTTTGGTTG
Plet1_F	CACTATGGCTAACGTCTCTGG
Plet1_R	CTGTCGTCCTCCTTCACTG
Plet1-001_F	GTCCTCATCGTCGTCAATC
Plet1-001_R	TCGTGTTTATGGTCTGGC
Plet1-002_F	CCGTGAAAATGGAACAAG
Plet1-002_R	CAGAGTTGACCAGAGTGAGTG
Prl2c-(Plf)_F	AACGCAGTCCGGAACGGGG
Prl2c-(Plf)_R	TGTCTAGGCAGCTGATCATGCCA
Prl3b1-(Pl2)_F	GCACTCGGGGAACAGCAGCC
Prl3b1-(Pl2)_R	ACTGCCAGCAACAGGAGTGCC
Prl3d1/2/3-(Pl1)_F	TTATCTTGGCCGCAGATGTGT
Prl3d1/2/3-(Pl1)_R	GGAGTATGGATGGAAGCAGTATGAC
Prl8a9_F	AAGAGAAAACTCCTGGAAGACC
Prl8a9_R	AACAATTTATAATGTTTGCCCTGTG
Rin3_F	ATTGGTGCTGTGTGTCCACT
Rin3_R	GCCTTCCAGGTACAGTATAGCC
Sdha_F	TGGTGAGAACAAGAAGGCATCA
Sdha_R	CGCCTACAACCACAGCATCA
Sox2_F	GAGTGGAAACTTTTGTCCGAGA
Sox2_R	GAAGCGTGTACTTATCCTTCTTCAT
Spry4_F	TCCTCAAAGACCCCTAGAAGC
Spry4_R	CATGACTGAGCTGGGATTCA
Tbp_F	GAGCTCTGGAATTGTACCGC
Tbp_R	GTTGTCCGTGGCTCTCTTATTC
Tead4_F	TCTAATGCCTTCTTCCTTGTGA
Tead4_R	GAGCAGACCTTCGTAGAGCA
Tfap2c_F	GCCGGACGCCATGTTGTGGA
Tfap2c_R	ACCCCGGTGTGCGAGAGAGG
Tfeb_F	TCAGAAGCGAGAGCTAACAG
Tfeb_R	TGCGTCTTCTCTCAATTAGGT
Tinagl1_F	TGATTCCAACGACATCTACCA
Tinagl1_R	CTTCCATGAGTGCTTGAACAG
Tpbpa_F	ACTGGAGTGCCCAGCACAGC
Tpbpa_R	GCAGTTCAGCATCCAACTGCG
Zic3_F	ACACTGGCGAGAAACCCTTC
Zic3_R	ACCGTCTGTCACAGCCTTC

**Bisulphite sequencing primers**	
Plet1 bis -1293F	GGTAAAATTTGTTTAGTTTTTAGGA
Plet1 bis -783R	CCAATACACTCCATACTTACCTTA
Plet1 bis -1270F	GAAGTTTTGGATTTAGTTATTAGTG
Plet1 bis -803R	TTACCTTAAATTAATTCTCTATACATTC
Plet1 bis -417F	TGAGATAAAAAGGGTATTTAAGTTTG
Plet1 bis -89R	CCTCCTAAAAACATTACCTTCTAAA
Plet1 bis -395F	TTTGAGTTTTTGATAGAGGAAAGTA
Plet1 bis -131R	ACCTAAATAAATCACCCAACCT
Plet1 bis -41F	GTTTTAGAGTATATAAATAGGAGTTTGTT

Plet1 bis 456R	CTTAACACAACCTTATATTACCAAAAT
Plet1 bis -1F	TATTATTGTTAGGTTTTGTAATTTAAATAG
Plet1 bis 420R	CTAAAACTCAATTTACCCCA

**Plet1 CRISPR gRNAs**				
**Name**	**Strand**	**Exon**	**target oligo (5'-3')**	**complementary oligo (5'-3')**
gRNA1	-	1st	CACCGATGTTATCAAGGACCACGC	AAACGCGTGGTCCTTGATAACATC
gRNA2	+	1st	CACCGACTATGGCTAACGTCTCTGG	AAACCCAGAGACGTTAGCCATAGTC
gRNA3	-	2nd	CACCGACTGACTGAATCGTTCACG	AAACCGTGAACGATTCAGTCAGTC
